# The application of GBS markers for extending the dense genetic map of rye (*Secale cereale* L.) and the localization of the *Rfc1* gene restoring male fertility in plants with the C source of sterility-inducing cytoplasm

**DOI:** 10.1007/s13353-016-0347-4

**Published:** 2016-04-16

**Authors:** Paweł Milczarski, Monika Hanek, Mirosław Tyrka, Stefan Stojałowski

**Affiliations:** 1 Department of Plant Genetics, Breeding and Biotechnology, West Pomeranian University of Technology in Szczecin, Słowackiego 17, 71-434 Szczecin, Poland; 2 Department of Biochemistry and Biotechnology, Rzeszów University of Technology, Powstańców Warszawy 6, 35-959 Rzeszów, Poland

**Keywords:** Cytoplasmic male sterility (CMS), Genetic map, Genotyping by sequencing (GBS), Rye

## Abstract

Genotyping by sequencing (GBS) is an efficient method of genotyping in numerous plant species. One of the crucial steps toward the application of GBS markers in crop improvement is anchoring them on particular chromosomes. In rye (*Secale cereale* L.), chromosomal localization of GBS markers has not yet been reported. In this paper, the application of GBS markers generated by the DArTseq platform for extending the high-density map of rye is presented. Additionally, their application is used for the localization of the *Rfc1* gene that restores male fertility in plants with the C source of sterility-inducing cytoplasm. The total number of markers anchored on the current version of the map is 19,081, of which 18,132 were obtained from the DArTseq platform. Numerous markers co-segregated within the studied mapping population, so, finally, only 3397 unique positions were located on the map of all seven rye chromosomes. The total length of the map is 1593 cM and the average distance between markers is 0.47 cM. In spite of the resolution of the map being not very high, it should be a useful tool for further studies of the *Secale cereale* genome because of the presence on this map of numerous GBS markers anchored for the first time on rye chromosomes. The *Rfc1* gene was located on high-density maps of the long arm of the 4R chromosome obtained for two mapping populations. Genetic maps were composed of DArT, DArTseq, and PCR-based markers. Consistent mapping results were obtained and DArTs tightly linked to the *Rfc1* gene were successfully applied for the development of six new PCR-based markers useful in marker-assisted selection.

**Electronic supplementary material:**

The online version of this article (doi:10.1007/s13353-016-0347-4) contains supplementary material, which is available to authorized users.

## Introduction

Rye (*Secale cereale* L.) is an outbreeding cereal grown in many regions of the world, but it is most popular in Central and Eastern Europe. This is due to its high resistance to the unfavorable environmental conditions frequently occurring in this area, such as sandy, acidic soils, frosty winters, and water deficiencies in spring. Rye is grown in farms applying modern, intense agricultural technologies, but tolerance to soils with a low content of nutrient solutions and moderate susceptibility to plant diseases also makes it very useful for low-input farming. Despite the importance of this crop in numerous European countries, breeding progress in rye is low compared to related cereals, for example wheat and barley. A noticeable acceleration in the development of new cultivars of rye has been achieved during the past three decades via the introduction of hybrid breeding methods (Geiger 2007).

Seed production of rye hybrid cultivars, similar to many other crops, is based on systems of cytoplasmic male sterility (CMS). The first documented case of CMS in rye was described in the United States by Putt (1954), but this source of sterility-inducing cytoplasm was likely not saved. Subsequently, beginning in the late 1960s, several reports on CMS in rye have been published (e.g., Kobyljanskij 1969; Geiger and Schnell 1970; Łapiński 1972; Madej 1975; Adolf and Winkel 1985). Currently, based on the results of test crosses (Warzecha and Salak-Warzecha 1996; Łapiński and Stojałowski 2003) and molecular analyses (Stojałowski et al. 2006, 2008), it is assumed that two genetically different cytoplasms inducing male sterility exist in rye. The Pampa sterility-inducing cytoplasm was discovered by Geiger and Schnell (1970) in a primitive Argentinean population, and this type of CMS is now the most significant for the development of hybrid cultivars of rye. The majority of the remaining reported cases of the discovery of CMS sources supposedly revealed the same genetic type of CMS, namely cytoplasm Vavilovii (CMS-V). These are represented by CMS sources designated as CMS-R (Kobyljanskij 1969), CMS-C (Łapiński 1972), CMS-G (Adolf and Winkel 1985), and others.

Nuclear genes that restore male fertility in rye with Pampa and Vavilovii cytoplasms are spread throughout the rye genome; rough mapping analyses have indicated that they are localized on chromosomes 1R, 3R, 4R, 5R, and 6R (Börner et al. 1998; Miedaner et al. 2000; Bednarek et al. 2002; Stojałowski et al. 2004). Among these genes, those most efficient at fertility restoration were located on the long arm of chromosome 4R. Significant progress in mapping the main restorer gene for the Pampa cytoplasm (*Rfp1*) has been achieved (Stracke et al. 2003; Hackauf et al. 2012); less information is available with respect to CMS-V restorers. Recently, the application of diversity array technology (DArT) for the construction of a high-density consensus map of rye based on recombinant inbred line (RIL) populations (Milczarski et al. 2011) enabled the identification of markers more closely linked to *Rfc1*, the main restorer gene for rye with CMS-C (Stojałowski et al. 2011).

This study was aimed at the application of next-generation sequencing (NGS) technology for genotyping by sequencing (GBS) to expand the genetic map of rye and, finally, localize the *Rfc1* gene with a higher level of precision.

## Materials and methods

### Plant material

For extending the genetic map of seven rye chromosomes, the mapping population “RIL-S” was applied. This population consisted of a set of RILs developed from a cross between two lines advanced in inbreeding: 541 and 2020LM. This mapping population had previously been analyzed during the construction of a high-density consensus genetic map of rye, and detailed information is given by Milczarski et al. (2011). The maternal line of the studied RIL population was a non-restorer for CMS-C, and the parental line 2020LM contained the *Rfc1* gene that restored plant fertility. The test crosses and following phenotyping analyses allowed for an assessment of which RIL carries the dominant restorer alleles and which possesses the recessive non-restorer allele; these analyses were previously described by Stojałowski et al. (2011).

The new mapping population was developed to localize the *Rfc1* gene. The source material for this population was a hybrid between male-sterile inbred line 544C containing the C cytoplasm and the restorer inbred line Ot0-20. Research conducted on the F_2_ generation of this hybrid indicated that the paternal line contained at least two effective restorer genes and that one of the supposed genes was located on the 4RL chromosome (Stojałowski et al. 2004). The male-fertile hybrid plants from the F_1_ and following generations were used for consecutive back-crossing with male-sterile line 544C. In the BC_5_ generation, eight male-fertile plants were selfed. As a result, a BC_5_F_2_ mapping population consisting of 1194 individuals was obtained. Plants of the mapping population were grown with 25 × 25 cm spacing in an experimental field located at Szczecin. Before flowering, 3–5 spikes of each individual plant were isolated with the use of bags made of transparent breathable foil. During flowering, each plant was visually assessed 2–3 times with respect to pollen shedding, and observations were recorded by using the nine-step scale suggested by Geiger and Morgenstern (1975). The correctness of visual observations was verified after harvest by the assessment of seed setting in previously isolated spikes. Individuals with inconsistent phenotyping results were removed from further analyses.

### Generation of molecular markers

Within both studied mapping populations, three methods were applied for the generation of molecular markers:

1. Polymerase chain reaction (PCR): two types of sequence-specific markers were used: sequence characterized amplified regions (SCARs) and simple sequence repeats (SSRs). Analyses of these markers within the RIL-S population had previously been used for the construction of a consensus genetic map, so all important methodological information can be found in Milczarski et al. (2011) and Stojałowski et al. (2009).
2. DArT: the microarray-based analyses were performed as a commercial service offered by Diversity Arrays Technology Pty Ltd. (Canberra, Australia), and the methodological procedure for the development of DArT markers in rye is described by Bolibok-Brągoszewska et al. (2009).
3. DArTseq: the platform developed by Diversity Arrays Technology Pty Ltd. for high-throughput genotyping based on sequencing results generated by NGS technologies. This variant of the GBS method was optimized for numerous plant species and is offered as a commercial service by DArT P/L. The principles of this method were recently published by Li et al. (2015). In brief, the crucial point of the analytical procedure is the method of reduction of genome complexity developed initially for the DArT mentioned above. This reduction is achieved by the application of optimized combinations of restriction enzymes. Sequencing analyses are performed on a HiSeq2000 (Illumina, USA), and the results consist of two groups of detected markers based on short DNA sequences. The first group is designed as SNP markers containing detailed data about the type and position of mutations that enable detection of the heterozygotes and both types of homozygotes within the mapping population (so-called co-dominant markers). The second group is called Silico-DArT, whose data inform only about the presence or absence of a given sequence variant (these markers are of dominant character).

### Construction of a high-density genetic map of rye

The maximum level of deviation of the markers from the distribution of 1:1 characteristic of the RIL populations was set at *p* = 0.005. After the reduction of markers significantly deviated from the assumed model, those remaining were binned for reducing redundant markers using the QTL IciMapping software (Wang et al. 2014a) separately for Silico-DArT and SNP. Redundant markers are completely correlated in a population and cannot provide additional information. In the next step, markers with missing rates higher than 20 % were deleted. Markers that were selected as representatives of the bin and not assigned to the bins were combined with DArT markers, as described previously by Milczarski et al. (2011). The genetic map construction was conducted using Multipoint 3.2 software (Ronin et al. 2012). The marker groups were formed at a maximum threshold level of recombination frequencies at 0.05 using the “order” command. For testing the reliability of multilocus mapping by detection and removing problematic markers that caused neighborhood instabilities, “control of monotony” was used. Finally, the ordering was repeated.

In the mapping population, we found an increase in the length of genetic maps compared to the original consensus map constructed using low-density genotyping data. The map length for a chromosome with a minimum distance between 1000 markers ≥1 cM provides a map of ~1000 cM. This is unrealistic in the vast majority of organisms, but it is unlikely to be caused by high genotype calling error rates. Wang et al. (2014b) suggested that it could be a result of the accumulation of low levels of genotyping error in maps which contain many thousands of markers. For reducing the inflation of genetic distances on a high-density genetic map, the average length of the consensus map (Milczarski et al. 2011) was used for the proportional scaling of obtained linkage groups, as previously described by Cavanagh et al. (2013) and Maccaferri et al. (2014).

### Comparative mapping and localization of the *Rfc1* gene on a higher resolution map

Identification of molecular markers tightly linked to the *Rfc1* gene was performed within both studied mapping populations using JoinMap 3.0 software (Van Ooijen and Voorrips 2001). The data concerning all segregating markers (PCR-based, DArT, DArTseq) were analyzed (redundant markers were not excluded from the analysis). Linkage groups were constructed at LOD = 20, and the group containing the *Rfc1* locus was selected for mapping in each population. Linkage maps were constructed for these two groups by using a regression mapping algorithm. Results were compared using MapChart 2.1 software (Voorrips 2002).

In order to increase the precision of mapping of the *Rfc1* gene, sequencing data about tightly linked DArT and DArTseq markers located on RIL-S and [544 × Ot0-20]BC_5_F_2_ mapping populations were used for the designation of PCR primers. These primers, as well as previously developed SCAR markers (Stracke et al. 2003; Stojałowski et al. 2011) located in the vicinity of the *Rfc1* gene, were applied for analyses on an extended number of individuals from the [544 × Ot0-20]BC_5_F_2_ population. In total, 658 individuals were genotyped to construct a map with increased resolution.

### Effectiveness of markers in the selection of non-restorer lines and DNA sequence annotations

CMS-C is not widely applied for breeding rye hybrids, mainly due to the deficiency of non-restorer lines and their stable male-sterile analogs. Currently, only a few related non-restorer lines are available for studies. We chose a set of 95 inbred lines developed from a cross between non-restorer 541 and restorer Ot1-3 as plant material for testing the effectiveness of marker-assisted selection (MAS). All these lines were test-crossed and their ability to restore or not restore male fertility in the CMS-C system was determined in field experiments (Stojałowski et al. 2011). PCR-based markers from the above higher resolution map, which revealed polymorphism between 541 and Ot1-3, were applied for genotyping of the 95 studied inbred lines and for the hypothetical selection of non-restorer lines. The effectiveness of selection was verified using a standard Chi^2^ test. Markers were considered efficient when the ratio of non-restorer and restorer lines differed significantly from that observed within the total population of studied lines (48:47).

The known DNA sequences of markers used for mapping the *Rfc1* gene were functionally annotated by performing a Blast search using Blast2GO 3.0 software (https://www.blast2go.com/) (Conesa et al. 2005). Blast matches were considered significant with E-values < 1e^−3^ conducted against the non-redundant (nr) NCBI sequence database. Gene Ontology (GO) annotations were performed to retrieve molecular function, biological process, and cellular component terms. The sequences were loaded into the Blast2GO program, and BLAST with a minimum E-value of 10^−3^ was performed with the program prior to mapping. The mapping step allowed the annotation of the sequences to the GO database for GO terms.

## Results

### Results of high-throughput genotyping methods

Both mapping populations were investigated by using two high-throughput genotyping technologies: DArT and DArTseq. The results of DArT analyses of the RIL-S mapping population had previously been used for construction of the consensus genetic map of rye (Milczarski et al. 2011). The applied methods of marker generation were very efficient: each type was represented by thousands of markers (Table 1). Among these, significant numbers revealed polymorphisms within the RIL-S population. The most numerous group of markers were the Silico-DArTs. They were almost twice as efficient with respect to the detection of genetic polymorphisms as SNP markers developed by using the same platform (DArTseq). The less numerous group of markers polymorphic within the RIL-S population was obtained with the use of the microarray method named DArT. On the other hand, over 2000 of the markers generated by this method covered the whole rye genome and was sufficient for the development of linkage groups of all seven rye chromosomes.

**Table 1:** Number of markers obtained from diversity array technology (DArT) and genotyping by sequencing (GBS) analyses within the two analyzed mapping populations

Population	541 × 2020LM RIL (RIL-S)			544 × Ot0-20 BC_5_F_2_		
Type of marker	DArT^a^	Silico-DArT	SNP	DArT	Silico-DArT	SNP
Total number of markers	11,514	39,908	14,888	3739	40,280	27,120
Number of polymorphic markers	2257	23,437	4417	348	3215	1237
Frequency of polymorphic markers	19.6 %	58.7 %	29.7 %	9.3 %	8.0 %	4.6 %

^a^Data from DArT analysis within the RIL-S population have been previously published (Milczarski et al. 2011)

The level of polymorphism detected within the [544 × Ot0-20]BC_5_F_2_ population was significantly lower than that observed in RIL-S population (Table 1), which was expected as an effect of several cycles of back-crossing performed during development of the mapping population. In this plant material, the SNP markers obtained from the DArTseq platform were also less numerous than the Silico-DArTs. The classic DArT technique based on hybridization of microarrays generated in the [544 × Ot0-20]BC_5_F_2_ population the smallest group of polymorphic markers, similarly to that recorded in RIL-S.

### High-density genetic map of rye

**Fig. 1:**
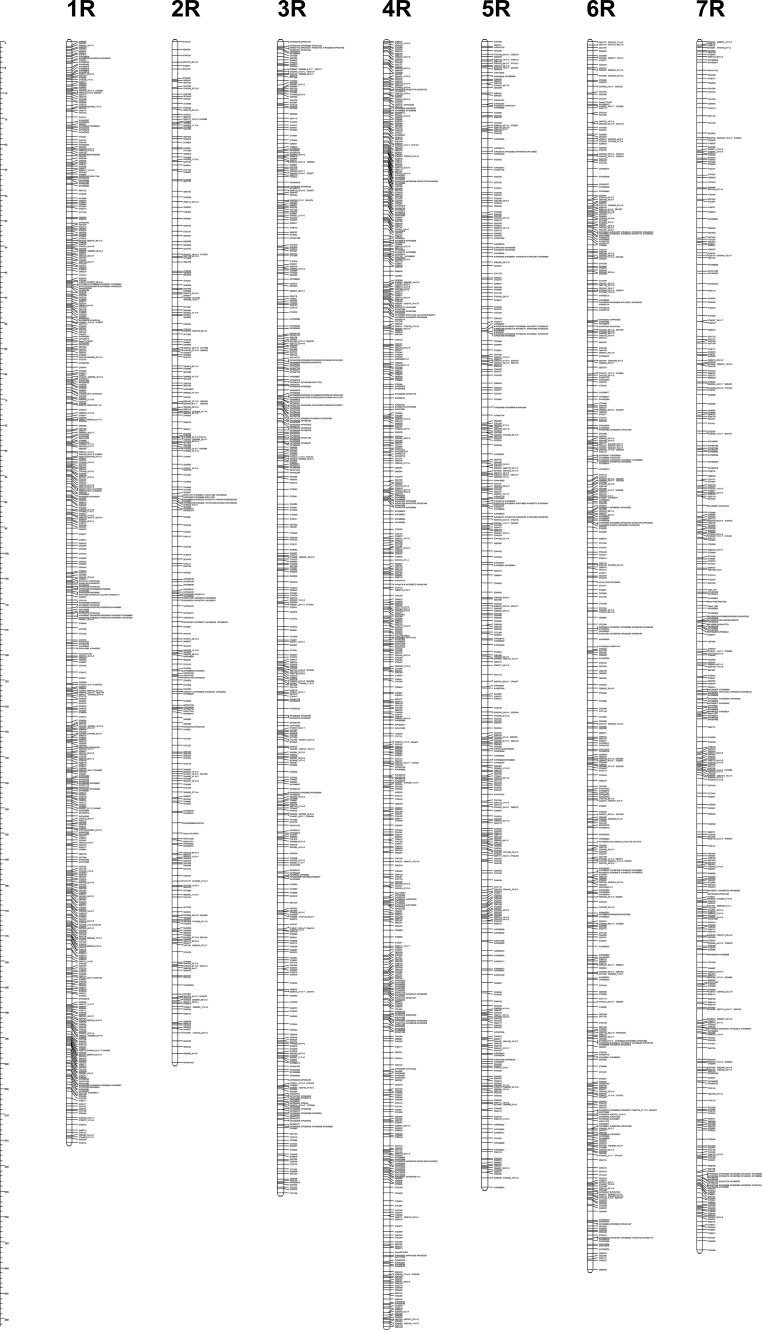
Genetic map of the RIL-S population (the scale on the left margin shows distances in cM)

The previously released genetic map of the RIL-S population (Milczarski et al. 2011) was expanded via the addition of two types of newly developed GBS markers: Silico-DArTs and DArTseq SNPs. During the mapping procedure from the group of more than 30,000 polymorphic markers (Table 1), numerous markers were not included into linkage groups. Finally, the current version of the map contains more than 19,000 markers (Table 2). The majority of Silico-DArT and SNP markers revealed identical distributions within the mapping population. These “redundant” markers were located in the same position (the same locus) and were grouped within bins represented on the map by one member of each bin. Finally, almost 3400 loci were revealed on the map (Fig. 1). Chromosomes consist of between 300 and almost 600 loci. When the data from bins are considered, the numbers of markers assigned to chromosomes vary from 1878 on 2R to 3812 on 6R (Table 2). The majority of these markers are Silico-DArTs.

**Table 2:** Characteristics of the rye genetic map developed for the RIL-S population

Chromosome	Number of markers assigned to chromosomes					The length of the map (cM)^b^	Average distance between loci (cM)	Max. distance between loci (cM)
	DArT	Silico-DArT^a^	SNP^a^	PCR-based	Total^a^			
1R	131	1970 (363)	421 (83)	5	2527 (581)	215.50	0.371	1.41
2R	70	1605 (194)	202 (57)	1	1878 (322)	199.90	0.621	2.02
3R	165	2463 (272)	207 (53)	0	2835 (490)	225.20	0.460	1.53
4R	168	1816 (357)	273 (61)	7	2264 (593)	251.40	0.424	1.74
5R	112	1763 (242)	293 (69)	0	2168 (423)	224.30	0.530	1.88
6R	180	3022 (270)	606 (98)	4	3812 (552)	240.20	0.435	2.03
7R	102	2632 (275)	859 (55)	4	3597 (436)	236.50	0.542	2.26
Total	928	15,271 (1972)	2861 (476)	21	19,081 (3397)	1593.00	0.469	2.26

^a^In brackets: markers shown on the genetic map of RIL-S (number of loci located on the map), remaining markers were included into bins and are not visualized on the map (these are localized within the loci represented by one marker from each bin)

^b^The length of the recalculated map (as described in the Materials and methods section; more information is available in Supplementary Table ESM1)

Markers on the genetic map are distributed relatively evenly along all seven chromosomes (Fig. 1). The shortest map was constructed for chromosome 2R (about 200 cM), and this was also the map with the lowest coverage by marker loci; there are only 322 loci mapped on this chromosome (Table 2). The average distance between loci mapped on 2R slightly exceeds 0.6 cM. Linkage maps of the remaining chromosomes contain at least a hundred more loci, and their lengths vary from 215.5 (1R) to 251.40 (4R). The longest gap between marker loci on this genetic map of rye is located on the 7R chromosome; it was estimated at 2.26 cM (Table 2).

**Fig. 2:**
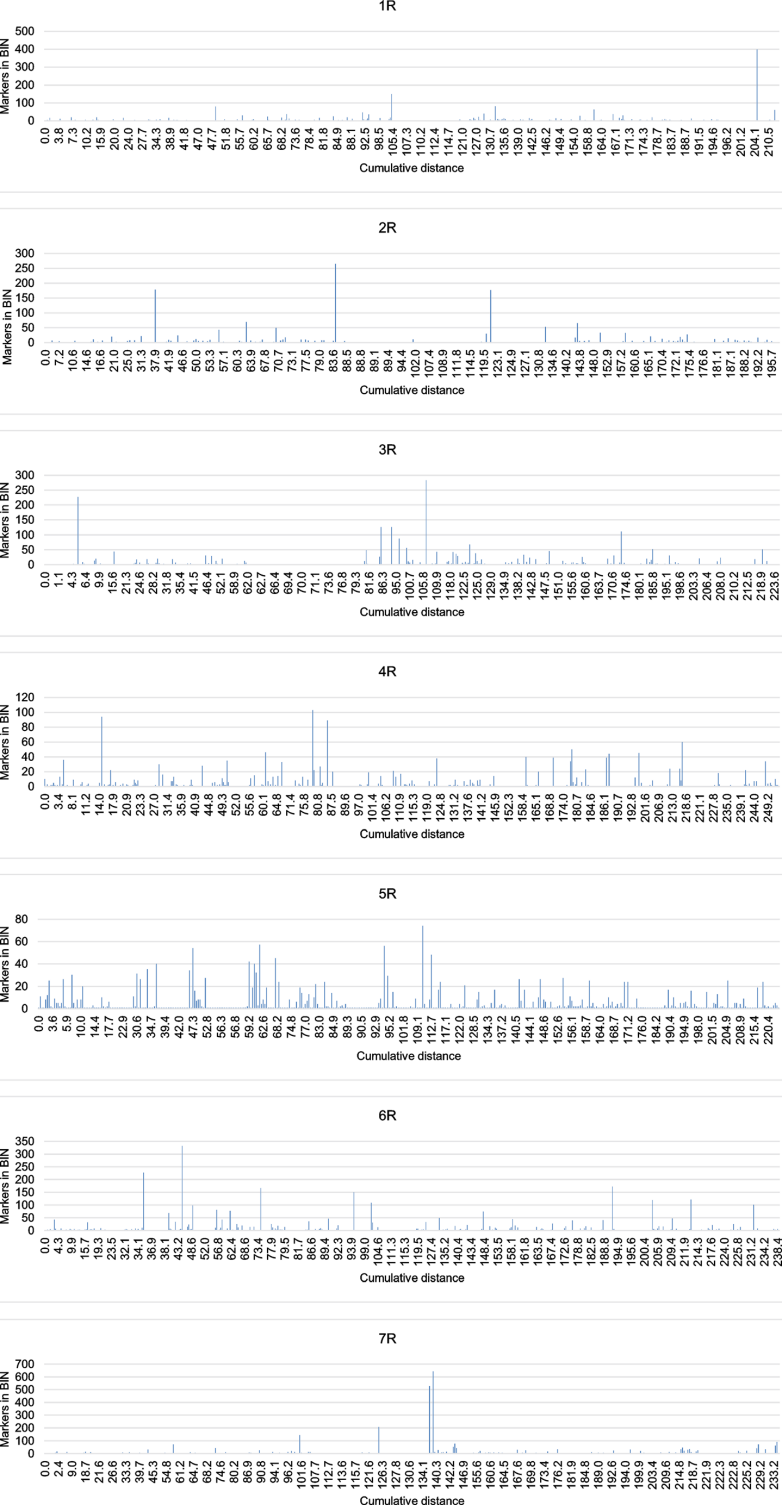
Distribution of markers within bins located on all rye chromosomes. The *x*-axis shows cumulative distance on the chromosome. The *y*-axis presents the number of markers included in the bin

Detailed data about the constructed genetic map containing positions of marker loci and their segregations within population RIL-S are given in the supplementary material ESM1. Among loci identified during map construction, a group of 1123 (about 33 %) contained more than one molecular marker and, on the map (Fig. 1), these are represented by only one of the binned markers. Redundant markers revealing polymorphisms, but not shown on the final map (included into bins), are listed in the ESM2 file. The distribution of binned loci containing more than one molecular marker is shown in Fig. 2. Two bin loci carrying the most numerous groups of markers (642 and 527) were found near each other, and they were mapped onto the proximal part of the 7R chromosome (Fig. 2). The largest bins located on the remaining chromosomes contained between 74 (5R) and 399 (1R) markers. In general, the location of loci abundant in co-segregating markers combined into bins seems to be randomly distributed: bins containing numerous markers could be found on proximal and distal parts of all rye chromosomes.

### Localization of the *Rfc1* gene and predicted efficiency of new markers for the selection of non-restorer lines

**Fig. 3:**
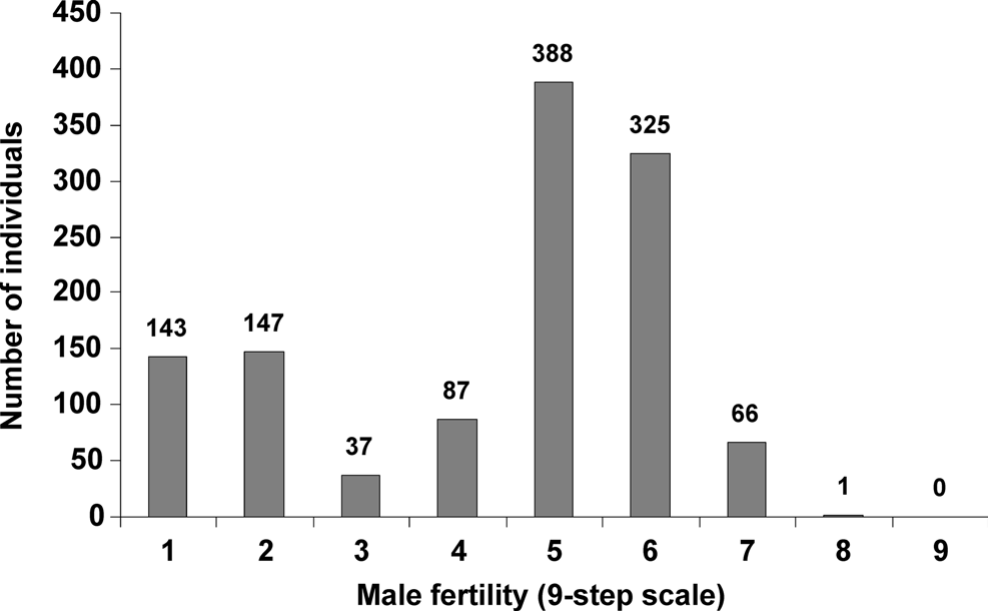
Distribution of male sterility/fertility within the studied mapping population [544 × Ot0-20]BC_5_F_2_

Phenotypic assessment within the studied population of [544 × Ot0-20]BC_5_F_2_ revealed a bimodal distribution of the trait. The largest groups, with more than 300 individuals, were those classified as partially male-fertile: 5 and 6 on the Geiger and Morgenstern (1975) scale. The next numerous classes, 1 and 2, include male-sterile plants (Fig. 3). When the whole population was divided into two main phenotypic classes, male-sterile (1–3 on the scale) and male-fertile (4–9 on the bonitation scale), the obtained amounts of individuals, 327:897, did not differ significantly from the expected 1:3 ratio (Chi^2^ = 3.63), likely indicating monogenic control of male fertility restoration.

One restorer gene was also found when the RIL-S population was studied. Phenotypic data have been published previously (Stojałowski et al. 2011), and the *Rfc1* gene was located on the genetic map of the long arm of chromosome 4R, at the 221 cM position (Fig. 1).

In the population [544C × Ot0-20]BC_5_F_2_, four groups of linked markers were identified at LOD = 20 (for details, see the ESM3 file). On the basis of the aforementioned map of the RIL-S population, these linkage groups were assigned to fragments of chromosomes 2R, 4R, 6R, and 7R. The most numerous group of markers was identified as a part of the 4R chromosome and it contained the *Rfc1* locus.

In order to increase the precision of mapping the part of the 4RL chromosome containing the *Rfc1* gene, the groups of markers tightly linked with this gene were selected for both analyzed populations: RIL-S and [544 × Ot0-20]BC_5_F_2_. The groups used for map construction consisted of 154 and 170 loci, respectively. Twenty-three markers present in these linkage groups were common for both populations: 21 DArTs and two SCARs (Xscsz23L500 and Xscsz670L900). No common marker from the DArTseq platform was identified.

**Fig. 4:**
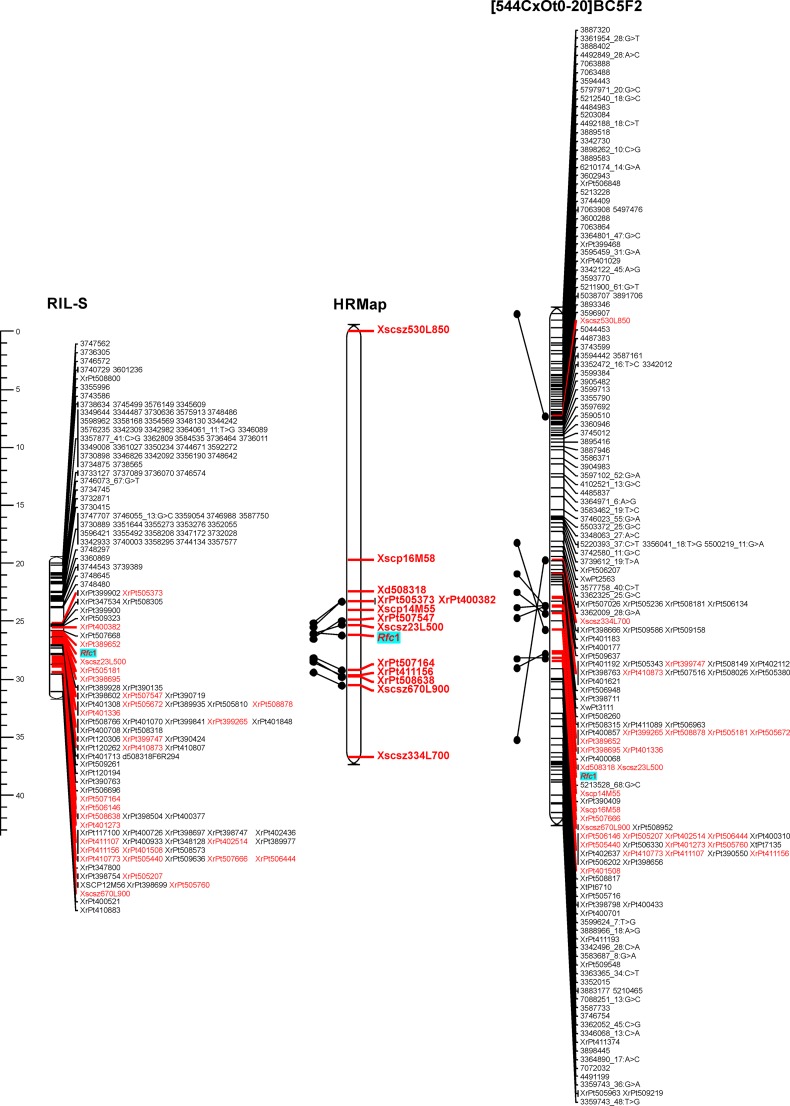
Comparative mapping of the genomic region containing *Rfc1*, the gene that restores male fertility in rye with CMS-C (the scale on the left margin shows distances in cM; markers commonly mapped within both studied mapping populations are printed in red; HRMap: genetic map based on analyses of extended numbers of individuals from the [544 × Ot0-20]BC_5_F_2_ mapping population; more details are given in the text)

For the construction of linkage maps covering the region of the genome carrying the *Rfc1* gene, all markers from the aforementioned linkage groups were applied (including co-segregating markers that, during development of the whole genetic map of the RIL-S population, were combined into bins). Loci identified in the RIL-S population are distributed within a distance of 11 cM (Fig. 4), and the *Rfc1* gene is located in the central part of this short linkage group between the DArT marker XrPt389652 and the SCAR marker Xscsz23L500. The map created for the second population covers a longer fragment of the 4R chromosome; markers are distributed along a distance of 43.5 cM (Fig. 4). Here, the *Rfc1* gene is also located in the middle part of the linkage map (position 25 cM), near markers Xscsz23L500 (SCAR) and 5213528_68:G>C (SNP).

On the basis of available DNA sequences related to DArT and DArTseq markers tightly linked with *Rfc1*, a set of 15 and 16 primer–pair combinations were designed for the development of PCR-based markers (Table ESM4). Of these, only six revealed polymorphism within the mapping population [544 × Ot0-20]BC_5_F_2_: XrPt400382, XrPt411156, XrPt505373, XrPt507164, XrPt507547, and XrPt508638. All these PCR markers were originally located on the genetic map as DArTs. Unfortunately, none of the markers based on DArTseq technology was successfully converted into a polymorphic PCR marker.

Analyses performed on the extended mapping population of the [544 × Ot0-20]BC_5_F_2_ cross resulted in the construction of the genetic map based on segregation analyses of 658 individuals of the mapping population which were conducted with the use of 13 PCR-based markers. Among these markers, ten were mapped within a distance not exceeding 5 cM from the *Rfc1* (Fig. 4). The remaining three markers were localized at a greater distance from the target gene and proved to be helpful for comparative mapping and have allowed the correct orientation of a map with higher resolution (HRMap). Six tightly linked markers with three newly converted from DArTs into PCR-based characters (XrPt507547, XrPt400382, and XrPt505373) were located in the proximal side of the *Rfc1* gene. On the distal part of the 4RL chromosome, the next three new PCR-based markers were mapped, but, here, the distance between *Rfc1* and the closest marker exceeded 2.5 cM.

Assessment of the utility of the PCR-based markers from the HRMap in terms of the identification of non-restorer lines and elimination of restorers was performed on a set of 95 inbred lines developed from one interline cross. From the group of ten markers located within a distance not exceeding 5 cM from *Rfc1*, seven revealed polymorphism between parental lines 541 and Ot1-3. None of these markers was perfect in the identification of genotypes of interest. On the other hand, analyses performed on the studied lines demonstrated the high efficiency of all seven tested markers in terms of the reduction of the group of 47 restorer genotypes; the number of restorers non-detected by a given marker varied from 4 to 9 (Table 3). Application of each marker for selection also led to the loss of some non-restorer lines. For the most effective markers, the number of false-negative indications did not exceed 10 % (four lines from the group of 48). As the studied markers were located on both proximal and distal sides of the *Rfc1* gene on the HRMap, it could be predicted that simultaneous application of a combination of markers should increase the efficiency of selection. Indeed, when all seven markers were analyzed simultaneously, false-positive indications (restorers classified by MAS as non-restorers) were reduced to only one case, and the same was noticed for false-negative results; one non-restorer line was included on the basis of genotyping into a group of restorer lines. Detailed data on the genotyping of the 95 studied inbred lines are presented in Table ESM5.

**Table 3:** Identification of non-restorers and restorers for the CMS-C system within the total population of recombinant inbred lines derived from the hybrid 541 × Ot1-3 and after selection with the use of the mentioned molecular markers (significance of marker-assisted selection assessed by the Chi^2^ test)

Marker	Non-restorer	Restorer	Total	Significance of MAS (Chi^2^)
XrPt411156	44	6	50	28.09**
XrPt507164	44	5	49	30.23**
XrPt508638	44	5	49	30.23**
XrPt400382	41	4	45	29.65**
XrPt505373	41	4	45	29.65**
Xscsz23L500	29	7	36	12.99*
Xscp14M55	33	9	42	13.22*
Total population	48	47	95	

*Statistically significant at *p* = 0.0005

**Statistically significant at *p* = 0.0001

Available DNA sequences of DArT and DArTseq markers used for mapping the *Rfc1* gene (Fig. 4) were searched within databases in order to detect relationships with known gene sequences. The results of detected annotations are presented in the ESM6 file. For the majority of analyzed sequences, no relationships with sequences deposited in databases were found. Within the remaining markers, similarity to genomic sequences of wheat and *Brachypodium distachyon* was revealed. The functions of the indicated genes originating from these grass species do not seem to be related to male fertility restoration in rye.

## Discussion

The first genetic map of all seven rye chromosomes was established more than 20 years ago, mainly based on restriction fragment length polymorphism (RFLP) markers (Devos et al. 1993). Subsequently, different marker systems have been utilized for mapping rye chromosomes, for example, RAPD (Masojć et al. 2001; Milczarski et al. 2007; Stojałowski et al. 2009), AFLP (Bednarek et al. 2003), and SSR (Hackauf and Wehling 2003; Khlestkina et al. 2004; Stojałowski et al. 2009; Myśków et al. 2010) markers, but only the application of DArT—the first available high-throughput method of genotyping *S. cereale*—created the possibility to construct high-density genetic maps of this species (Bolibok-Brągoszewska et al. 2009; Milczarski et al. 2011). Sequence analyses of RNA generated by Haseneyer et al. (2011) provided the opportunity for the development of the next high-throughput marker technology: the SNP arrays successfully used for the study of the structure of the rye genome as well as for genetic mapping (Martis et al. 2013). NGS methods were recently used for the development of GBS technologies that have led to the highly efficient and cost-effective discovery of SNPs in different species (Kumar et al. 2012). The high numbers of SNPs identified during a single analysis can be applied for precise assessment of genetic diversity, construction of linkage maps, and detection of markers linked with genes controlling important agronomic traits. The DArTseq platform used in this study collates the sequence analysis results in two sets of markers: dominant Silico-DArT and co-dominant SNP markers. The first group is more numerous, the second more informative (allowing for distinguishing heterozygotes from dominant homozygotes and indicating the exact character and position of mutations within the DNA molecule). Recently, GBS markers generated by this platform have been successfully applied for the construction of genetic maps of *Brassica carinata* (Zou et al. 2014), watermelon (Ren et al. 2015), and wheat (Li et al. 2015). It should be stressed, however, that alternative methods of GBS have also been developed and have proven their effectiveness, for example, in mapping cereal genomes, such as barley and wheat (Poland et al. 2012) or rubber tree (Pootakham et al. 2015).

During genetic map construction, one of the supposed advantages of the application of SNP markers discovered by NGS technologies is the high polymorphism relatively evenly distributed within the whole genome (Kumar et al. 2012). Application of this method for extending the genetic map of the rye genome confirms this suggestion. Implementation of GBS markers (Silico-DArTs and DArTseq SNPs) into the genetic map of rye (Milczarski et al. 2011) has resulted in a significant enhancement of map density and elimination of gaps exceeding the distance of 5 cM (Fig. 1; Table ESM1). This agrees with the results of Wendler et al. (2014), who reported the efficiency of GBS technology for the precise identification of even exceptionally small introgressions from wild into cultivated species. These authors performed their experiments on one of the most important and thoroughly studied self-fertile cereals, barley, but suggested that their statements should be of universal character and applicable for breeding activities in any crop.

The results from the localization of the *Rfc1* gene on the long arm of the 4R chromosome (Fig. 4) were compared with data from a newly developed mapping population [544C × Ot0-20]BC_5_F_2_. The cross combination between inbred lines 544C and Ot0-20 has previously been used for the identification of RAPD markers linked to restorer genes (Stojałowski et al. 2004), and a high level of pollen shedding within the studied F_2_ population was noticed. In the currently analyzed population, the genetic diversity was significantly reduced by several cycles of back-crossing. This resulted in a lower frequency of polymorphic markers (Table 1), but due to multiple recombination events in the region containing the *Rfc1* gene, this increased the precision of mapping. Phenotypic variation within this population was relatively narrow; the most male-fertile plants did not occur (Fig. 3). The lower pollen shedding by genetically fertile plants was probably due to inbreeding depression; plants of the studied population were genetically and phenotypically similar to maternal line 544C. An additional factor that could have led to a lack of fully fertile plants within the population was elimination during back-crossing of additional, less efficient, and still unrecognized restorer genes (Stojałowski et al. 2004). Compared linkage maps from the fragment of the 4RL chromosome were very well adjusted in both the RIL-S and [544C × Ot0-20]BC_5_F_2_ populations. Surprisingly, no common marker was identified from the DArTseq platform. All common markers belonged to the microarray DArT group and PCR-based markers. On the other hand, the compared linkage maps were relatively short, with limited numbers of markers, and it has been observed for three wheat mapping populations that the DArTseq platform frequently generates markers which are polymorphic within a single population (Li et al. 2015). Conversion of DArT and DArTseq markers into PCR-based markers was only partly successful. PCR primers designed on the basis of short known sequences of DArTseq markers produced exclusively monomorphic amplification products in this experiment. From our other research (not published), we know that sequence data obtained during GBS analyses sometimes allow for designation of primers revealing polymorphism within mapping populations, but the frequency of such cases is low. Available sequences of DArT clones were more efficient for designation of PCR-based markers; about 30 % of these proved to be useful for mapping and MAS.

Rye is still being extensively studied in genetic research. The first high-density maps started to be published at the end of the first decade of the 21st century (Bolibok-Brągoszewska et al. 2009; Milczarski et al. 2011) and were based on mapping populations with a limited number of analyzed genotypes not exceeding the range of 150. Recently published results of mapping the *Rfp1* gene that restores male fertility in the Pampa sterilizing cytoplasm were achieved with the use of 506 plant genotypes (Hackauf et al. 2012). A similar number of individuals (658) was used for the development of our HRMap. An increased number of genotyped plants resulted in some rearrangements of the map order. Of these, the most significant was the change of the position of the Xscsz334L700 marker. Its “floating” position on the map of population [544C × Ot0-20]BC_5_F_2_ could be a result of mapping algorithms and the fact that the *Rfc1* locus and the majority of markers located on the map near this gene are dominant markers segregating, in the so-called “coupling phase”, dominant alleles originating from the paternal Ot0-20 line. PCR products of the Xscsz334L700 marker originate from the maternal 544C line, so this marker remains in “repulsion phase” to almost all loci of the HRMap. Interestingly, one of the PCR markers, Xscp14M55, developed by Stracke et al. (2003), is present on our HRMap and a genetic map with the *Rfp1* gene investigated by Hackauf et al. (2012). On both genetic maps, it is located proximally from the main restorer genes interacting with two genetically different cytoplasms, indicating that *Rfp1* and *Rfc1* are located in the same region of the 4RL chromosome.

The potential applicability of PCR-based markers localized in the vicinity of the *Rfc1* gene for the selection of non-restorer genotypes seems to be high. All markers located close to the target gene were statistically effective. Of course, the precision of selection by using any studied marker is not perfect and, ultimately, traditional phenotyping is still needed, but frequencies of non-restorers and restorers can be significantly changed before this time and labor-intensive activities are initiated. The efficiency of MAS can be additionally increased by the use of 2–3 markers simultaneously. On the other hand, one should stress that male fertility restoration in the CMS-C system remains under the control of more than one gene (Stojałowski et al. 2004) and development of perfect molecular markers for the selection of non-restorer or restorer genotypes needs the identification of additional, less effective restorer genes present in the rye genome. In the population [544C × Ot0-20]BC_5_F_2_, in spite of five cycles of back-crossing, heterogeneous regions were found not only on 4R, but also on 2R, 6R, and 7R chromosomes. However, these three chromosomes are probably not engaged in the control of male fertility in the studied CMS system. Restorer genes have never been localized on chromosomes 2R and 7R. Only on 6R it was suggested the presence of some weak restorers for CMS-P (Miedaner et al. 2000; Bednarek et al. 2002) and CMS-C (Stojałowski et al. 2004), but their localizations were not certain.

In conclusion, the genetic map of the rye genome with the *Rfc1* gene localized on the 4RL chromosome reported here reveals the power of high-throughput marker technologies based on microarrays and NGS applications for mapping important genes. The results of mapping the *Rfc1* gene indicate that this gene is located in the vicinity of the *Rfp1* gene that restores male fertility in rye with genetically different CMS-Pampa (Hackauf et al. 2012).

However, it should be mentioned that high-density genetic maps presented in this study are not true high-resolution maps. The relatively low numbers of individuals genotyped within both studied mapping populations still does not allow for the construction of highly precise maps. Especially in the RIL-S population, which consisted of less than 100 individuals, this resulted in the presence of many bins containing numerous markers located at the same locus (Fig. 2). On the other hand, to our knowledge, this is the first report concerning the application of GBS markers (obtained from the DArTseq platform) for mapping rye chromosomes, and even if the resolution of the map is not very high, the presented database of markers anchored to particular chromosomes should significantly facilitate utilization of this high-throughput marker technology for further study of the rye genome.

## Electronic supplementary material

Below are the links to the electronic supplementary material.

ESM 1 (XLSX 1003 kb)

ESM 2 (XLSX 271 kb)

ESM 3 (XLSX 23 kb)

ESM 4 (XLSX 10 kb)

ESM 5 (XLSX 12 kb)

ESM 6 (XLSX 35 kb)
